# Mediation Analysis of Racial Disparity for Infant Mortality Using Bayesian Estimation of Potential Outcomes

**DOI:** 10.3390/jcm13123464

**Published:** 2024-06-13

**Authors:** James Thompson

**Affiliations:** College of Veterinary Medicine and Biomedical Science, Texas A&M University, College Station, TX 77843-4475, USA; jthompson@cvm.tamu.edu

**Keywords:** causal inference, Bayesian, mediation modeling, infant mortality, potential outcome

## Abstract

**Background/Objectives**: While the overall rate of infant mortality in the United States has been decreasing over decades, the racial disparity, defined as the difference between races, has increased. Even though a person’s race cannot change, it may be possible to identify factors that mediate or cause this racial disparity. Evaluating the factors that mediate or cause racial disparity is imperative because current clinical recommendations could be based on preventative modalities that are more effective for white women and their children. **Methods**: A Bayesian approach modeled the data from the full United States National Natality Database for the years 2016 to 2018. The binomial rate parameters for each combination of race and mediators provided the potential outcomes. Estimating the mediation outcomes, including total effect, controlled direct effect, mediated effect, and proportion mediated used common counterfactual definitions for these probabilities. **Results**: Maternal smoking, low birthweight, and teenage maternity interacted in causing racial disparity for infant mortality. The proportion of racial disparity attributable to low birthweight was approximately 0.73, with only small variations attributable to maternal smoking and teenage maternity. **Conclusions**: The novel approach facilitated modeling of multiple mediators. Low birthweight caused racial disparity for infant mortality. The model can be extended to evaluate additional mediational factors with the objective of identifying the preventable causes.

## 1. Introduction

In the United States, Black infants are twice as likely as white infants to die in their first year of life. While the overall rate of infant mortality has been decreasing over several decades, the racial disparity, defined as the difference between races, has increased [1]. Recently, the National Institute on Minority Health and Health Disparities (NIMHD) supported a special issue that focused on the causes of racial health disparities in the United States [2,3]. The authors concluded that novel methods are necessary to identify causes whose manipulations could form the bases of preventive care programs [4,5]. While race itself cannot be manipulated, it may be possible to identify factors that mediate or cause racial disparity. The possibility that clinical recommendations could be based on preventive modalities that have been more effective for white women and their children makes evaluating this potential imperative. The scientific approach to identifying the sub-component causes of the racial disparity and to evaluating possible structural racism in health programs is called mediation modeling [6]. Evaluating multiple mediators has two severe limitations. First, the causal pathway among all variables, which is usually uncertain, must be specified, and second, with many causal pathways, more estimates are required than the data allow [7]. The causes of racial disparity are largely unknown but virtually certain to be multifactorial [1]. What is needed is a mediation model that can model interactions among multiple mediating causes.

This study’s objective was to implement and evaluate a simple Bayesian approach to model three well known causes of infant mortality as potential mediators that could interact in causing racial disparity. The evaluated mediators were maternal smoking, low birthweight, and teenage maternity. The goal was to perform mediation modeling by implementing direct Bayesian estimation of potential outcomes for the approximately 11 million observations from the United States National Natality Database for the years 2016 to 2018. An introduction to Bayesian modeling is available for members of the clinical audience who are unfamiliar with the Bayesian approach that also explains the technical language of Bayesian implementation [8]. 

## 2. Materials and Method

### 2.1. Data 

The source of data was the National Vital Statistics System which provides the data online (https://www.cdc.gov/nchs/nvss/births.htm, accessed on 7 June 2024). In the United States, state laws require birth certificates for all births, and federal law mandates national collection and publication of births and other vital statistics data. The National Vital Statistics System, the federal compilation of these data, is the result of the cooperation between the National Center for Health Statistics (NCHS) and the states to provide access to statistical information from birth certificates. The website also describes standard forms for the collection of the data and model procedures for the uniform registration of the events. Data from birth certificates are linked to data from death certificates for all deaths before one year of age. Data for the study were publicly available with all patient identifiers removed. The most recent three years of available data, including all births from 2016 to 2018 were downloaded at the start of the study. Analysis was restricted to singleton births. Infant mortality was the outcome and defined as death before one year of age. Race was the cause of interest (X) and classified as “Black” for all children born to a mother of Black ethnicity and “Non-Black” for all other ethnicities and races. The potential mediators included maternal smoking (M1; whether the mother reported smoking during pregnancy), low birthweight (M2; defined as less than 2.5 kg) and teenage maternity (M3; defined as maternal age less than 20 years). 

### 2.2. Model 

**Figure 1:**
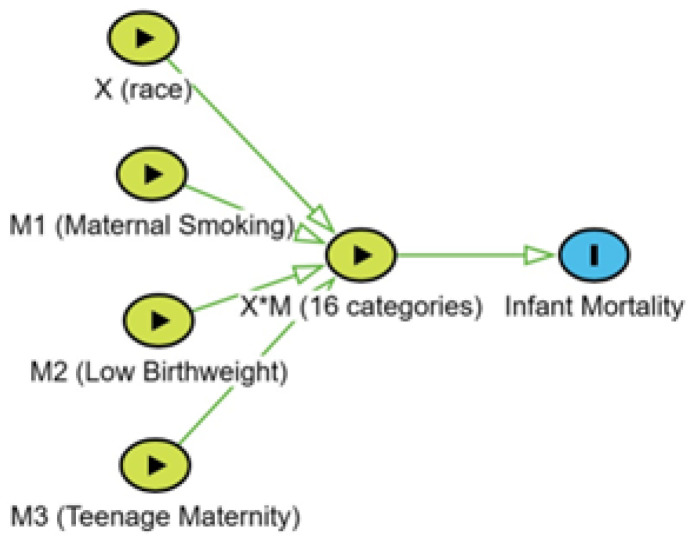
Directed Acyclic Graph. Each of the four binary nodes has a single arrow to the interaction node, and the interaction node has 16 classification levels.

The modeling was guided by a Directed Acyclic Graph (DAG) that contained a node representing interaction among a cause (X) and three mediators (M1, M2, and M3). The interaction node was a 16-level multivariate node representing the disease rates for all possible combinations of the cause and mediators that are referred to as potential outcomes (Figure 1). 

The model estimated rates for potential outcomes using direct Bayesian estimation. The data contained i = 16 rows of data with each row identified by unique values for X and three mediators (M1, M2, and M3). Each row contained a count of infant deaths (r_i_) and births (n_i_). The count of deaths r_i_ was modeled as binomial with a rate parameter PO[X_i_, M1_i_, M2_i_, M3_i_] and the count of births n_i_:

r_i_~Binomial(PO[X_i_, M1_i_, M2_i_, M3_i_], n_i_). (1)

The rate parameters were the potential outcomes and given Uniform(0,1) priors:

PO[1:2,1:2,1:2,1:2] ~ Uniform(0,1).(2)

For comparing two sets of risk factors, the relative risk was the ratio of the two potential outcomes and the risk difference was the difference between the potential outcomes. The full distribution of relative risk and risk difference was estimated by Markov Chain Monte Carlo.

Estimating the total effect (TE) used the standard counterfactual definition:

Prob(Y)|X = 2 − Prob(Y)|X = 1.(3)

Estimation of controlled direct effects (CDE) used standard counterfactual definitions for individual mediators and all possible combinations of mediators (Table 1). 

The proportion attributable (PA) was the difference between total effect and controlled direct effect expressed as a proportion of total effect:

PA = (TE − CDE)/TE.(4)

Minimally informative prior values were specified to avoid influencing the posterior distributions. The prior for each potential outcome was an equal probability for the entire range of 0 to 1. Estimation was performed using MultiBUGS 1.0 [9]. The data and code suitable for MultiBUGS are provided in Appendix A. A burn-in of 5000 iterations was discarded, and the next 10,000 iterations were collected for posterior distributions. Convergence was determined by monitoring chains with disparate starting values. Reported results are the median and 95% credibility intervals which were the 2.5 and 97.5 percentiles taken directly from the posterior distributions. Goodness-of-fit among models was compared using the Deviance Information Criterion [10].

## 3. Results

The records identified 11,226,394 singleton births and 54,808 infant deaths before one year of age for an overall infant mortality rate of 0.0049 (0.0048, 0.0049). Maternal race was not missing for any of the births. The mortality rate among children born to women of Black ethnicity was 0.0089 (0.0088, 0.0090), and the mortality rate among children born to women of other ethnicities and races was 0.0041 (0.0040, 0.0041). The estimate of the risk difference between the two rates is the total effect of racial disparity which was an additional risk probability of 0.0048 (0.0047, 0.0050) for children of Black mothers. The corresponding relative risk was 2.18 (2.14, 2.22). The portion of this total effect on racial disparity mediated by low birthweight was 0.73 (0.71, 0.75), and the portion of the total effect for racial disparity mediated by all three mediators combined (maternal smoking, low birthweight, and teenage maternity) was 0.74 (0.72, 0.76). There was little mediation of racial disparity for maternal smoking and teenage maternity and maternal smoking combined with teenage maternity (Table 2). 

The goodness of fit among the models was evaluated using the Deviance Information Criterion [10], and the best fitting model included all interactions among race and the three mediators (Table 3).

The modeling results are based on the 16 outcome probabilities, defined by combinations of race, smoking, low birthweight, and teenage maternity (Table 4).

## 4. Discussion

The causal-inference literature has reported considerable methodological developments in mediation analysis over the last ten years, arguably providing the basis for the novel methods called for by the NIHMD [7]. The advancements include mathematical tools for mediation modeling that are based on theoretical counterfactuals often referred to as alternative outcomes. These changes are claimed to enable valid causal inferences from observational data [11]. Originally, the approach was limited to the evaluation of causal variables only at specific levels of the mediating variable [12]. This reasoning was accepted and advanced by modeling and interpreting the interaction, thereby estimating the causal effect not at one level of the mediator but at all levels in an approach based on structural equation modeling (SEM) [7]. However, estimating the variance of functions utilizing estimates from separate equations continues to be problematic in the frequentist approaches. Bayesian SEM implemented using Markov chain Monte Carlo estimation accounts for asymmetrical (non-normal) distribution of effects [13]. The Bayesian implementation of the SEM approach that includes interaction between the cause and the mediator shows promise for implementing and interpreting single cause and single mediator models, but evaluating multiple mediators has severe limitations [7].

Counterfactual modeling has addressed the limitation in frequentist thinking that a modeler cannot know a patient’s alternative outcome that would have occurred if the facts had changed and the mediator and other causes under consideration had been different. An alternative direct Bayesian approach that does not include counterfactual considerations has long been proposed [14,15,16]. In Bayesian thinking, the condition of disease versus non-disease for an individual is the product of a probability process. It is the probability of the process, not the factual outcome, that is important in the analysis. Conditional upon assumptions and measurable uncertainty, all patients with a matching vector of causes have the same probability of disease. Should the causes change, a single patient’s alternative outcome is the estimated probability of disease in the new stratum defined by X (race) and the new mediators (M). In Bayesian analyses, each patient has a current probability of disease, and if the causes were to change, a counter probability of disease. The expression “counterfactual” is not appropriate for Bayesian analyses, but the expression “potential outcome” is. Under a Bayesian model with potential interactions among the cause and multiple mediators, the risk probabilities for all combinations of cause and mediators are directly estimable and constitute the full set of potential outcomes. 

In the United States, for the three-year period 2016 to 2018, the infant mortality rate for singleton births was 0.0048 (0.0048, 0.0049). The mortality rate among children born to women with Black ethnicity was more than twice that among children born to women of other races and ethnicities. The magnitude of this risk is well known, but the causes are poorly understood [1]. The current study provides insight into the cause of this disparity in that a proportion of 0.73 (0.71, 0.75) of the total effect was explained by low birthweights. Maternal smoking and teenage maternity modified this proportion, but only slightly. Reporting that low birthweight causes racial disparity for infant mortality does not directly identify manageable causes, but these results demonstrate a novel approach that facilitates a path forward for identifying strategies to prevent this racial disparity.

The strengths of the study include both flexible model specification and direct estimation of potential outcome probabilities. In mediation modeling, it is now common practice to include interactions among the cause of interest and potential mediators [7,12]. However, causal diagrams to model effect modification have been inadequate [17,18]. Weinberg proposed representing the effect modification by crossing arrows (Figure 1 in Weinberg) [17]. Attia et al. proposed two further modifications [18]. The first was to add a node at the point of crossing arrows, and the second was to link additional causes as also having an arrow to both the interaction node and the outcome (Figures 2b and c in Attia et al.) [18]. The current study could have extended the Attia et al. DAG to have four binary nodes (X, M1, M2, and M3), with each node having one arrow to infant mortality and a second arrow to an eight-level interaction node. The current study’s DAG (Figure 1) is computationally equivalent but has the advantage of exploiting the extraordinary simplicity of estimating the full set of 16 potential outcomes using Bayesian estimation. The graphical model specifies potential confounding among all binary variables (X, M1, M2, and M3) because of a common downstream variable (the interaction X*M). The causal effects of X, M1, M2, and M3 are on the interaction node. An important distinction of the model is that race is not causally responsible for the mediators of racial disparity. This is a highly desirable modification of models for investigating racial disparity. 

Direct estimation of potential outcome probabilities with Bayesian modeling provides a distinct advantage over frequentist approaches. Bayesian analysis addresses the probability of parameters like racially specific risks, while frequentist analysis addresses the likelihood of collecting data as extreme as the observed data. This fundamental difference in approaches has motivated frequentist modelers to make additional assumptions and manipulate multiple parameters to derive probability estimates. This study estimated 16 potential outcomes (Table 4). Individual patients can use differences among potential outcomes to compare expected results should the mediators change. For multiple mediator models, the frequentist estimation process for the complete set of direct and indirect effects is too complex without extraordinary assumptions [7,19,20,21,22,23,24]. In contrast, Bayesian estimation of the probability of Y (pY) at each level of interaction provides the full set of potential outcomes (pY|X,M1,M2,M3) and is extraordinarily simple for anyone comfortable with Bayesian modeling. The approach provides the same conditional probabilities as those estimated using stratification and provides full control for confounding among the included variables. Analyzing the data in tabular format will not be limited by the number of observations. Computer run time rather than the number of estimated distributions will limit the number of mediators. The introduction to MultiBUGS evaluated the run time for a model with 20,426 random effects and 425,112 observations and showed it was manageable on a personal computer [9]. 

The limitations of the study include the potential for omitted causes and classification of variables measured on a continuous scale. The assumption for the current study was that the data are conditionally exchangeable. Because the study used stratification for conditioning, the results assume that for each of the 16 potential outcomes, there is no more information that makes the observations within that cell correlated. A more useful description of the assumption would be that there were no causes of infant mortality omitted from any of the 16 cells in the tabulated data. While that is a simple assumption, Bayesian philosophy that it is likely that at least one cause is missing from any analysis of observational data should prevail. The Bayesian approach to specify a simple assumption that is virtually certain to be false has advantages over an obscure set of complex assumptions that an investigator encourages readers to accept. First, the assumption is easy for readers to understand and empowers them to consider and propose one or more missing causes. This should lead to further investigation, including the estimation of potential outcomes for more complex interactions. Such an investigation will often lead to a superior model. Results from the current study can illustrate this process. The study shows that, among the mediators evaluated, the best two-mediator model included maternal smoking and low birthweight (Table 3). Proposing that this two-mediator model could be missing teenage maternity as a cause is a useful criticism. Responding to the criticism by inserting teenage maternity into the interaction can evaluate whether teenage maternity contributes to dependence within the cells of the two-mediator model. Comparing the potential outcomes for teenage maternity = no (Table 4; row 1) and teenage maternity = yes (row 2) completes the evaluation. The table shows an increase in potential outcomes for Non-Black infants from 0.0014 (0.0014, 0.0015) to 0.0027 (0.0026, 0.0029) and for Black infants from 0.0027 (0.0026, 0.0028) to 0.0037 (0.0034, 0.0041). This shows dependence within these two strata and supports the inclusion of teenage maternity as a causal variable. Evaluating the other six interaction classifications provides little evidence that omitting teenage maternity from other combinations of maternal smoking and low birthweight would result in correlation within the cells. There are dozens of potential mediators, and the current model is certainly missing causal variables. However, the approach should enable a fuller investigation of multiple potential mediators.

Continuous variables are often converted to categorical variables by grouping values, but this can result in a loss of information, power, and efficiency [25]. The study’s objective was to estimate the full mediational potential for optimizing birthweights and used a binary classification, even though birthweight is usually categorized within five classes from extremely low birthweight to increased birthweight (macrosomia). Further investigation should identify any lost information. Some variables such as age, for example, are measured on a linear scale, but reducing the linear scale to multiple classes can be advantageous as an alternative to assumptions made regarding linearity [26].

The U.S. National Natality Database collects data from more than three million childbirths per year. Records contain dozens of potential mediators of racial disparity, including maternal, paternal, neonatal, and community factors. Until now, a lack of flexible mediation modeling approaches involving two or more mediators has been an obstruction. The current study promotes further mediation modeling by illustrating a novel approach and by reporting remarkable preliminary findings.

Besides different rates between the two race groups for both prevalence of mediators and race-specific risks, there is a difference in the acceptance of public health programs. A legacy of racial discrimination in medical research and the health care system has been linked to a low level of trust in medical research and medical care among African Americans [27]. This mistrust is associated with perceived discrimination, and addressing this perception is one of the key elements of addressing racial health disparities [28]. Clinical researchers should evaluate every preventive and treatment recommendation as a potential source of structural racism. Implementing the evaluation approach illustrated here will optimize clinical decisions and eventually mitigate racial mistrust.

## 5. Conclusions

Bayesian mediation modeling provided direct estimation of the target probabilities defined for current frequentist applications. The approach showed remarkable potential for evaluating mediating causes when there are multiple mediators. The results showed that low birthweight caused considerable racial disparity for infant mortality. Reporting that low birthweight causes racial disparity does not directly identify the preventable causes, but these results promote a novel path forward for identifying them. The proposed research approach permits the evaluation of multiple interacting mediators. There are approximately 50 potential causes of infant mortality, including maternal, paternal and social factors recorded in the database. Future research should seek to determine how these factors interact in causing racial disparity for infant mortality. 

## Figures and Tables

**Table 1:** Definitions of controlled direct effect (CDE). The counterfactual definition for CDE defines the probability of infant mortality P(Y) conditional upon specific values for race (X) and mediators M1 (smoking), M2 (low birthweight, LBW), and M3 (teenage maternity). The model estimator is the value from the model that estimates the CDE.

Mediator	Counterfactual Definition of CDE	Model Estimator of CDE ^1^
M1	P(Y)|X = 2, M1 = 1 − P(Y)|X = 1, M1 = 1	PO[2,1,NA,NA] − PO[1,1,NA,NA]
M2	P(Y)|X = 2, M2 = 1 − P(Y)|X = 1, M2 = 1	PO[2,NA,1,NA] − PO[1,NA,1,NA]
M3	P(Y)|X = 2, M3 = 1 − P(Y)|X = 1, M3 = 1	PO[2,NA,NA,1] − PO[1,NA,NA,1]
M1,M2	P(Y)|X = 2, M1 = 1, M2 = 1 − P(Y)|X = 1, M1 = 1, M2 = 1	PO[2,1,1,NA] − PO[1,1,1,NA]
M1,M3	P(Y)|X = 2, M1 = 1, M3 = 1 − P(Y)|X = 1, M1 = 1, M3 = 1	PO[2,1,NA,1] − PO[1,1,NA,1]
M2,M3	P(Y)|X = 2, M2 = 1, M3 = 1 − P(Y)|X = 1, M1 = 1, M3 = 1	PO[2,NA,1,1] − PO[1,NA,1,1]
M1,M2,M3	P(Y)|X = 2, M1 = 1, M2 = 1, M3 = 1 − P(Y)|X = 1, M1 = 1, M2 = 1, M3 = 1	PO[2,1,1,1] − PO[1,1,1,1]

^1^ PO[a,b,c,d] is the potential outcome for: a (race) equals 1 for Non-Black and 2 for Black; b (maternal smoking) equals 1 for no and 2 for yes; c (low birthweight) equals 1 for no and 2 for yes; d (teenage maternity) equals 1 for no and 2 for yes. A value of NA means the indicator value is treated as missing.

**Table 2:** Proportion of Effect Mediated. Estimates of the proportion of racial disparity attributable to smoking, low birthweight, teenage maternity, and combinations of these mediators.

Mediating Factor(s)	Proportion of Racial Disparity Attributed to the Mediating Factor(s)
Smoking	0.01 (−0.03, 0.05)
Low birthweight	0.73 (0.71, 0.75)
Teenage maternity	−0.00 (−0.05, 0.04)
Smoking and low birthweight	0.73 (0.71, 0.75)
Smoking and teenage maternity	0.02 (−0.03, 0.06)
Low birthweight and teenage maternity	0.73 (0.71, 0.75)
Smoking, low birthweight, and teenage maternity	0.74 (0.72, 0.76)

**Table 3:** Model Fit. Models are listed in order (top to bottom) of improving fit (lower Deviance Information Criterion indicates better fit).

Model	DIC ^1^	Effective Parameters
Intercept	127,700	1
Race	121,400	2
Race and teenage maternity	120,700	4
Race and smoking	118,300	4
Race, smoking, and teenage maternity	117,700	8
Race and low birthweight	3023	4
Race, low birthweight, and teenage maternity	2621	8
Race, smoking, and low birthweight	562	8
Race, smoking, low birthweight, and teenage maternity	171	16

^1^ DIC is the Deviance Information Criterion.

**Table 4:** Potential Outcomes. The potential outcome is the estimate of the probability of an infant’s mortality, based on the maternal/neonatal factors. The racial disparity can be assessed for all combinations of mediators. The relative risk is the ratio of potential outcomes and the risk difference is the difference betweeen potential outcomes.

Presence of Maternal/Neonatal Factors (Yes/No)			Probability of Mortality (Potential Outcome)	
Maternal Smoking	Low Birthweight	Teenage Maternity	Non-Black Infant	Black Infant
No	No	No	0.0014 (0.0014, 0.0015)	0.0027 (0.0026, 0.0028)
No	No	Yes	0.0027 (0.0026, 0.0029)	0.0037 (0.0034, 0.0041)
No	Yes	No	0.045 (0.044, 0.045)	0.057 (0.056, 0.058)
No	Yes	Yes	0.052 (0.050, 0.055)	0.056 (0.052, 0.059)
Yes	No	No	0.0044 (0.0042, 0.0046)	0.0071 (0.0065, 0.0077)
Yes	No	Yes	0.0054 (0.0047, 0.0061)	0.0069 (0.0045, 0.0097)
Yes	Yes	No	0.039 (0.038, 0.041)	0.051 (0.048, 0.055)
Yes	Yes	Yes	0.045 (0.039, 0.051)	0.056 (0.041, 0.073)

## Data Availability

The data for this study is provided in Appendix A.

## Appendix A. Data and MultiBUGS Code for Direct Bayesian Estimation of Potential Outcomes with 3 Mediators

model{for (i in 1:16){  r[i] ~ dbin(PO[X[i], M1[i], M2[i], M3[i]], n[i]) }
for (j in 1:2){for (k in 1:2){for (l in 1:2){ for (m in 1:2){ PO[j,k,l,m] ~ dunif(0,1) }}}}
for (j in 1:2){for (i in 1:16){ n.race[i,j] <- equals(X[i],j) * n[i] } #loop i end
                 sum.n.race[j] <- sum(n.race[ ,j]) }
for (j in 1:2){for (k in 1:2){for (l in 1:2){for (m in 1:2){
n.mv[m,j,k,l] ~ dbin(PF[m,j,k,l], sum.n.race[m])
PF[m,j,k,l] ~ dunif(0,1)  }
RRF[j,k,l] <- PF [2,j,k,l]/PF [1,j,k,l]
RRO[j,k,l] <- PO[2,j,k,l]/PO[1,j,k,l]   }}}
for (i in 1:16){  n.mv[X[i], M1[i], M2[i], M3[i]] <- n[i]
        r.mv[X[i], M1[i], M2[i], M3[i]] <- r[i] }
for (j in 1:2){for (k in 1:2){for (l in 1:2){
r.X.M1.M2[j,k,l] <- sum(r.mv[j,k,l,]); n.X.M1.M2[j,k,l] <- sum(n.mv[j,k,l,])
r.X.M1.M2[j,k,l] ~ dbin(PO.X.M1.M2[j,k,l], n.X.M1.M2[j,k,l])
PO.X.M1.M2[j,k,l] ~ dunif(0,1)
r.X.M1.M3[j,k,l] <- sum(r.mv[j,k,,l]); n.X.M1.M3[j,k,l] <- sum(n.mv[j,k,,l])
r.X.M1.M3[j,k,l] ~ dbin(PO.X.M1.M3[j,k,l], n.X.M1.M3[j,k,l])
PO.X.M1.M3[j,k,l] ~ dunif(0,1)
r.X.M2.M3[j,k,l] <- sum(r.mv[j,,k,l]); n.X.M2.M3[j,k,l] <- sum(n.mv[j,,k,l])
r.X.M2.M3[j,k,l] ~ dbin(PO.X.M2.M3[j,k,l], n.X.M2.M3[j,k,l])
PO.X.M2.M3[j,k,l] ~ dunif(0,1) }
r.X.M3[j,k] <- sum(r.X.M2.M3[j,,k]); n.X.M3[j,k] <- sum(n.X.M2.M3[j,,k])
r.X.M3[j,k] ~ dbin(PO.X.M3[j,k], n.X.M3[j,k])
PO.X.M3[j,k] ~ dunif(0,1)
r.X.M2[j,k] <- sum(r.X.M2.M3[j,k,]); n.X.M2[j,k] <- sum(n.X.M2.M3[j,k,])
r.X.M2[j,k] ~ dbin(PO.X.M2[j,k], n.X.M2[j,k])
PO.X.M2[j,k] ~ dunif(0,1)
r.X.M1[j,k] <- sum(r.X.M1.M2[j,k,]); n.X.M1[j,k] <- sum(n.X.M1.M2[j,k,])
r.X.M1[j,k] ~ dbin(PO.X.M1[j,k], n.X.M1[j,k])
PO.X.M1[j,k] ~ dunif(0,1) }
r.X[j] <- sum(r.X.M1[j,]); n.X[j] <- sum(n.X.M1[j,])
r.X[j] ~ dbin(PO.X[j], n.X[j]) 
PO.X[j] ~ dunif(0,1) }
r.total <- sum(r[])
n.total <- sum(n[])
r.total ~ dbin(m.rate, n.total)
m.rate ~ dunif(0,1)
TE <- PO.X [2] − PO.X [1]
RR <- PO.X [2]/PO.X [1]
CDE [1] <- PO.X.M1[2,1] − PO.X.M1[1,1]
CDE [2] <- PO.X.M2[2,1] − PO.X.M2[1,1]
CDE [3] <- PO.X.M3[2,1] − PO.X.M3[1,1]
CDE [4] <- PO.X.M1.M2[2,1,1] − PO.X.M1.M2[1,1,1]
CDE [5] <- PO.X.M1.M3[2,1,1] − PO.X.M1.M3[1,1,1]
CDE [6] <- PO.X.M2.M3[2,1,1] − PO.X.M2.M3[1,1,1]
CDE [7] <- PO[2,1,1,1] − PO[1,1,1,1]
for (i in 1:7){ ME[i] <- TE − CDE[i]; PA[i] <- 100*ME[i]/TE }}

Data

X[]	M1[]	M2[]	M3[]	n[]	r[]
1	1	1	1	7,877,239	11,253
1	1	1	2	365,117	999
1	1	2	1	422,306	18,817
1	1	2	2	289,50	1504
1	2	1	1	550,823	2422
1	2	1	2	37,682	201
1	2	2	1	70,523	2772
1	2	2	2	4591	205
2	1	1	1	1,453,357	3908
2	1	1	2	122,824	455
2	1	2	1	170,762	9680
2	1	2	2	17,473	974
2	2	1	1	81,169	575
2	2	1	2	3764	25
2	2	2	1	19,059	977
2	2	2	2	755	41

END

## References

[1] Jang C.J., Lee H.C. (2022). A Review of Racial Disparities in Infant Mortality in the US. Children.

[2] Alegria M., Araneta M.R., Rivers B. (2019). The National Advisory Council on Minority Health and Health Disparities Reflection. Am. J. Public Health.

[3] Alvidrez J., Castille D., Laude-Sharp M., Rosario A., Tabor D. (2019). The National Institute on Minority Health and Health Disparities Research Framework. Am. J. Public Health.

[4] Duran D.G., Perez-Stable E.J. (2019). Novel Approaches to Advance Minority Health and Health Disparities Research. Am. J. Public Health.

[5] Jeffries N., Zaslavsky A.M., Roux A.V.D., Creswell J.W., Palmer R.C., Gregorich S.E., Reschovsky J.D., Graubard B.I., Choi K., Pfeiffer R.M. (2019). Methodological Approaches to Understanding Causes of Health Disparities. Am. J. Public Health.

[6] Baron R.M., Kenny D.A. (1986). The Moderator Mediator Variable Distinction in Social Psychological-Research: Conceptual, Strategic, and Statistical Considerations. J. Pers. Soc. Psychol..

[7] VanderWeele T.J. (2015). Explanation in Causal Inference: Methods for Mediation and Interaction.

[8] Bayman E.O., Oleson J.J., Dexter F. (2024). Introduction to Bayesian Analyses for Clinical Research. Anesth. Analg..

[9] Goudie R.J.B., Turner R.M., De Angelis D., Thomas A. (2020). MultiBUGS: A Parallel Implementation of the BUGS Modeling Framework for Faster Bayesian Inference. J. Stat. Softw..

[10] Spiegelhalter D.J., Best N.G., Carlin B.P., van der Linde A. (2014). The deviance information criterion: 12 years on. J. Roy. Stat. Soc. B.

[11] Pearl J., Mackenzie D. (2018). The Book of Why: The New Science of Cause and Effect.

[12] Pearl J. (2014). Interpretation and Identification of Causal Mediation. Psychol. Methods.

[13] Miocevic M., Gonzalez O., Valente M.J., MacKinnon D.P. (2018). A Tutorial in Bayesian Potential Outcomes Mediation Analysis. Struct. Equ. Model..

[14] Dawid P. (2017). On individual risk. Synthese.

[15] Dawid A.P., Musio M., Fienberg S.E. (2016). From Statistical Evidence to Evidence of Causality. Bayesian Anal..

[16] Dawid A.P. (2000). Causal inference without counterfactuals. J. Am. Stat. Assoc..

[17] Weinberg C.R. (2007). Can DAGs clarify effect modification?. Epidemiology.

[18] Attia J., Holliday E., Oldmeadow C. (2022). A proposal for capturing interaction and effect modification using DAGs. Int. J. Epidemiol..

[19] Zugna D., Popovic M., Fasanelli F., Heude B., Scelo G., Richiardi L. (2022). Applied causal inference methods for sequential mediators. BMC Med. Res. Methodol..

[20] Tai A.S., Lin S.H. (2024). Multiply robust estimation of natural indirect effects with multiple ordered mediators. Stat. Med..

[21] Tai A.S., Lin S.H. (2021). Integrated multiple mediation analysis: A robustness-specificity trade-off in causal structure. Stat. Med..

[22] Steen J., Loeys T., Moerkerke B., Vansteelandt S. (2017). Flexible Mediation Analysis with Multiple Mediators. Am. J. Epidemiol..

[23] Loh W.W., Moerkerke B., Loeys T., Vansteelandt S. (2022). Disentangling indirect effects through multiple mediators without assuming any causal structure among the mediators. Psychol. Methods.

[24] Hao W., Song P.X.K. (2023). A Simultaneous Likelihood Test for Joint Mediation Effects of Multiple Mediators. Stat. Sin..

[25] Altman D.G. (1991). Categorizing Continuous-Variables. Brit J. Cancer.

[26] Thompson J.A. (2019). Disentangling the roles of maternal and paternal age on birth prevalence of down syndrome and other chromosomal disorders using a Bayesian modeling approach. BMC Med. Res. Methodol..

[27] Boulware L.E., Cooper L.A., Ratner L.E., LaVeist T.A., Powe N.R. (2003). Race and trust in the health care system. Public Health Rep..

[28] Bazargan-Hejazi S., Ruiz M., Ullah S., Siddiqui G., Bangash M., Khan S., Shang W., Moradi P., Shaheen M. (2021). Racial and ethnic disparities in chronic health conditions among women with a history of gestational diabetes mellitus. Health Promot. Perspect..

